# Supplementary material for: Photosynthesis-dependent H2O2 transfer from chloroplasts to nuclei provides a high-light signalling mechanism
Source: Nat Commun. 2017 Jun 29;8:49. doi: 10.1038/s41467-017-00074-w (PMC5491514; doi:10.1038/s41467-017-00074-w)
Supplement: Supplementary file 1 — Supplementary Information [file 41467_2017_74_MOESM1_ESM.pdf]

File name: Supplementary Information
Description: Supplementary Figures and Supplementary Tables

File name: Peer Review File
Description:

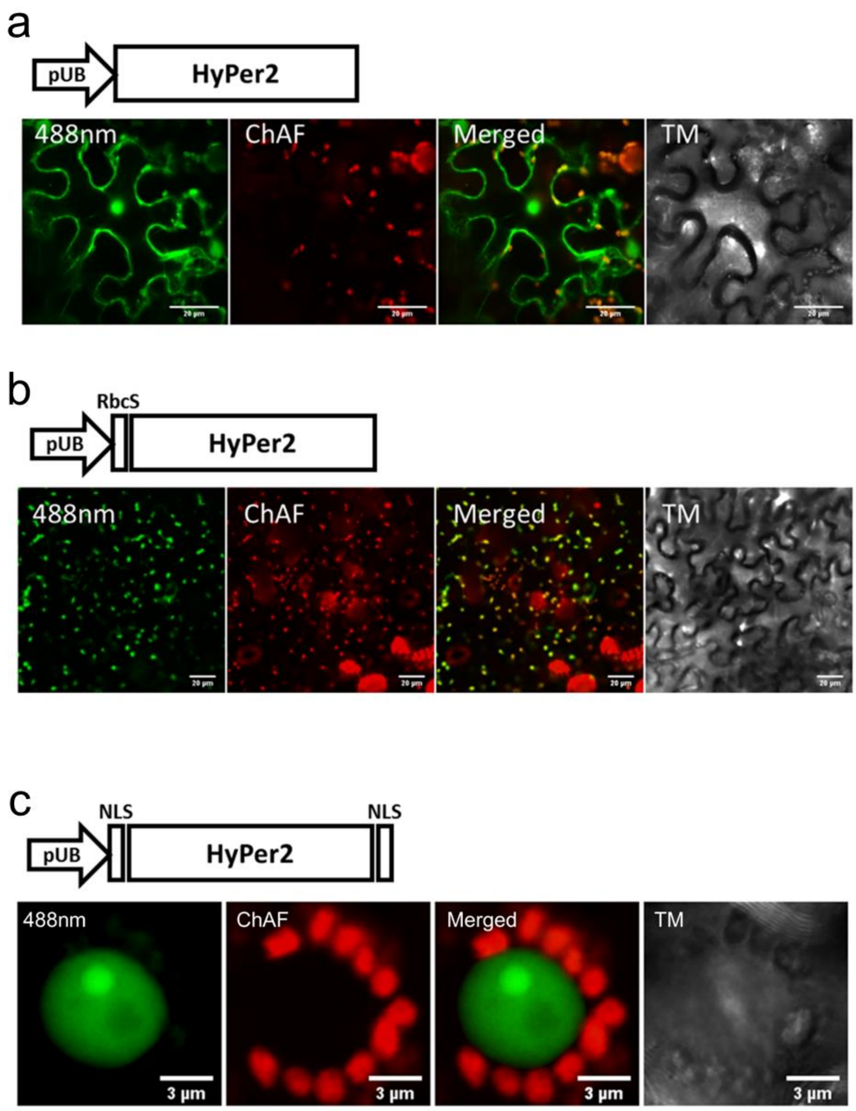

Supplementary Figure 1. Subcellular targeting of HyPer2 probes. Representative images of HyPer2 fluorescence in the cytosol and nuclei (**a**), chloroplasts (**b**) and nuclei only (**c**). Schematic diagrams of HyPer2 chimeric genes used are shown above each figure; pUB is the Ubiquitin gene promoter, RbcS is the small unit Rubisco subunit chloroplast transit peptide sequence and NLS is a nuclear localisation sequence. For full details of the constructs see Methods. Images in each column of panels from the left are as follows: HyPer2 fluorescence at 530nm from 488 nm excitation light, chlorophyll auto-fluorescence (ChAF), overlaid images of HyPer2 fluorescence and ChAF (merged) and from reflected light (TM). Scale bar 20 µm (**a**,**b**) and 3 µm (**c**)

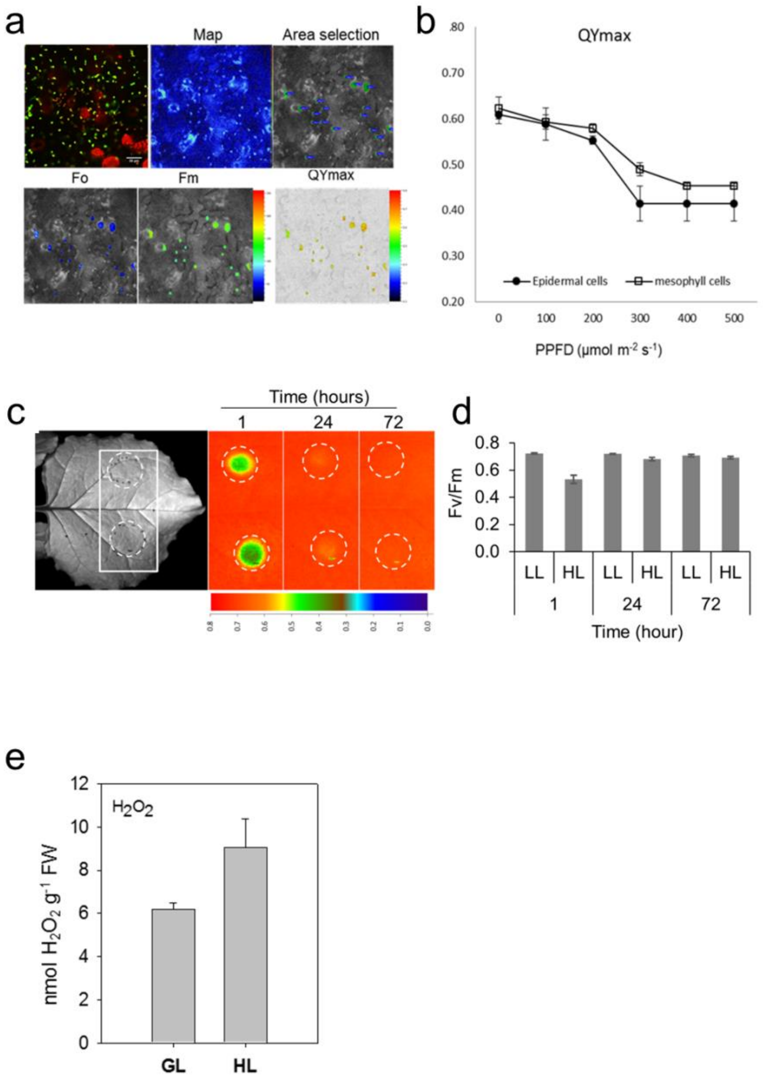

Supplementary Figure 2. High resolution chlorophyll fluorescence imaging of abaxial epidermal and mesophyll cells and the response of *N. benthamiana* leaves to HL. (**a**) High resolution chlorophyll fluorescence (CF) imaging to determine the maximum operating quantum efficiency (F_v'/F_m') of photosystem II using a PSI Micro-FluorCam. The images are as follows: Top left panel, 488 nm and 680 nm CF to show epidermal and mesophyll cell chloroplasts, in green and red respectively; top middle panel, map (reflected light) image to establish the focal plane; top right panel shows area and manual chloroplast selections for quantitative imaging (blue arrows). Bottom panels show collected and calculated quantitative CF parameters, F_0' (bottom left), F_m' (bottom middle) and QY_{max} (equivalent to F_v'/F_m'; bottom right). See methods and Baker (64) for further details of CF parameters. (**b**) F_v'/F_m' (QYmax) changes in epidermal and mesophyll cells in response to increasing light intensity (photosynthetically active photon flux density; PPFD). The values are the means ± SD (n= 15-25 epidermal chloroplast and 3-5 areas showing mesophyll chloroplasts) from two independent experiments. (**c**) Measurement of maximum dark-adapted quantum efficiency (Fv/Fm) by CF imaging of a *N. benthamiana* leaf following a HL exposure (1000 µmol m^{-1} s^{-1} PPFD) for 1 h on two areas of leaf (indicated by dotted circles). Fv/Fm was imaged after HL exposure and then 24 and 72 h after the plant was placed back in GL (120 µmol m^{-1} s^{-1} PPFD). The rectangle shown in the left panel image indicates the area from which Fv/Fm values were obtained for sectors of leaf not exposed to HL and are shown in panel d. (**d**) Fv/Fm data from two experiments of the type described in (c). (**e**) Foliar H_2O_2 concentration in GL exposed leaves and after 1h HL exposure. Determinations were made from acid extracts using an Amplex Red-based assay.

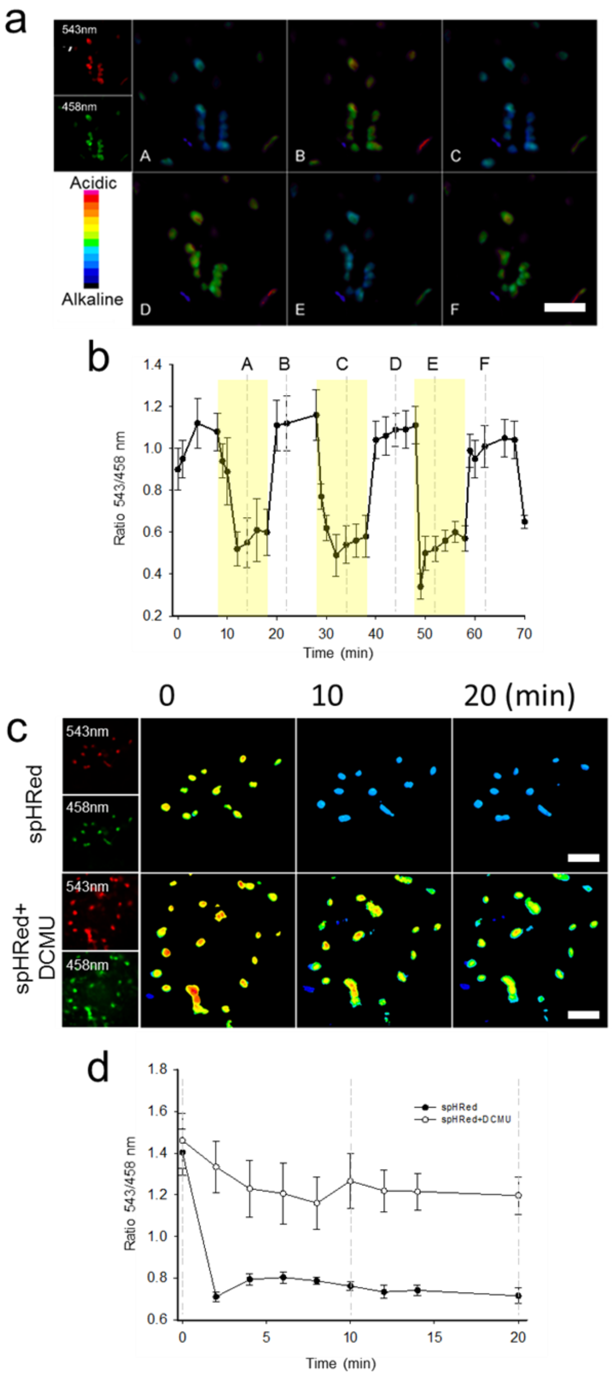

Supplementary Figure 3. Dynamics of pH changes in the stroma of *N. benthamiana* epidermal cell chloroplasts in response to GL and HL and its dependency on photosynthetic electron transport. pHRed was used to detect pH changes in chloroplast stroma. An increase in its 458/543 nm fluorescence ratio indicates an increase in pH. (**a**) Images of fluorescence (458/543 nm) changes of spHRed in alternating 10 min periods of dark, GL and finally HL. The timing of the images in panels A-F are shown in the graph in (b). (**b**) Quantification of the 458/543 nm fluorescence ratio during the alternating dark and light periods from 20 chloroplasts. Values are the means ± SEM. The points A-F are for reference to the images in (a). (**c**) Images of the 458/543 nm fluorescence ratio of spHRed going from dark to GL conditions in leaves treated with 10 µM DCMU or a water control. Zero-time data were collected immediately after switching on the light source. (**d**) Quantification of the 458/543 nm fluorescence ratio in chloroplasts from (c). The values are the means ± SE from 12 and 18 chloroplasts from a control and DCMU-treated leaf respectively. Scale bar = 20 µm.

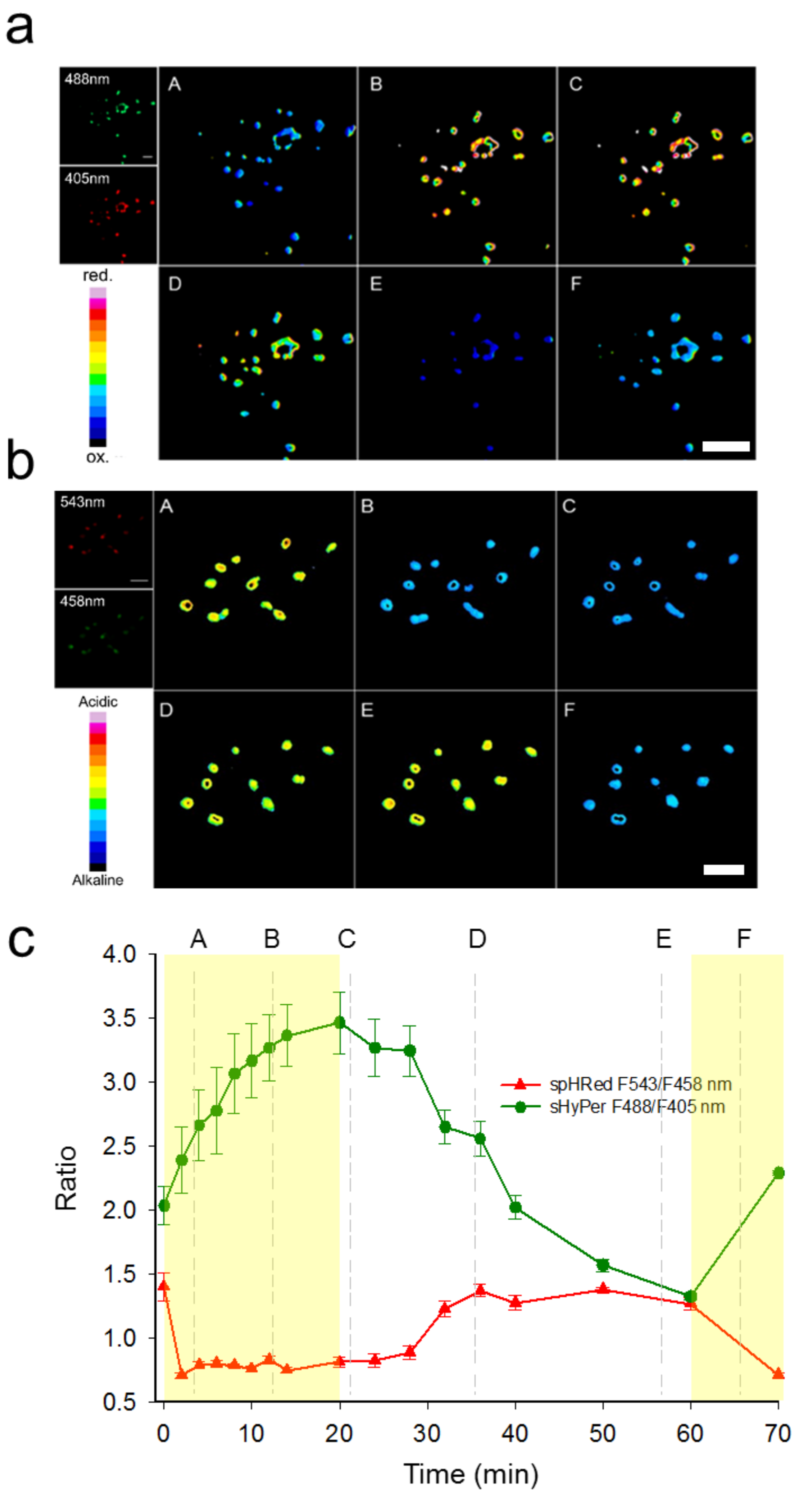

Supplementary Figure 4. Co-expression of sHyPer2 and spHRed in the stroma of *N. benthamiana* epidermal cell chloroplasts exposed to a HL and dark period. (**a**) and (**b**) Images of sHyPer2 (a) and spHRed (b) 405/485 nm and 458/543 fluorescence respectively in dark incubated leaves exposed to 20 min HL followed by a 45 min dark period and then re-exposed to HL. The timing of the images in the panels A-F is shown in the graph in (c). Scale bar = 50 µm. (**c**) Quantification of sHyPer2 and spHRed fluorescence in response to HL for 20 min, dark for 45 min and a subsequent repeat HL exposure. The points A-F are for reference to the images in (a) and (b). The values are the means (± SEM) from 20 and 25 chloroplasts for sHyPer2 and spHRed respectively from two independent experiments. Scale bar = 20 µm.

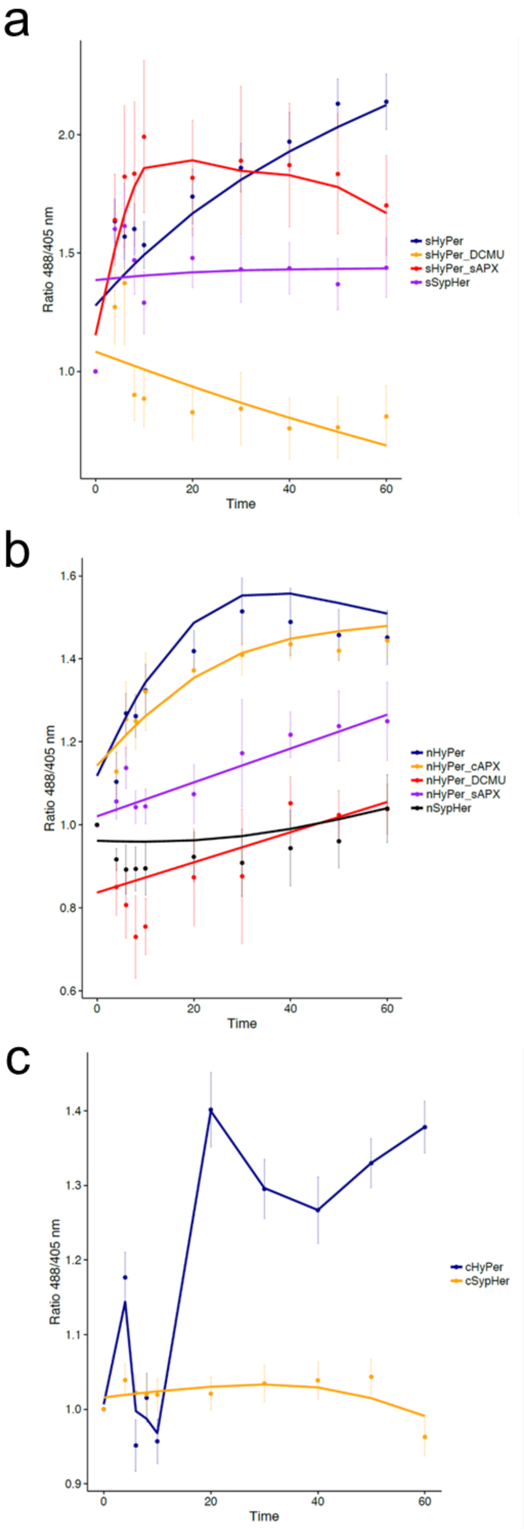

Supplementary Figure 5. Response curves of 488/405 nm fluorescence ratio to HL fitted using a generalised additive mixed effects model (GAMM). (a-c**)** figures of show the response curve fitted to ratio values shown in Figure 2. The complete GAMM multi-model selection is shown in Supplementary Table 1.

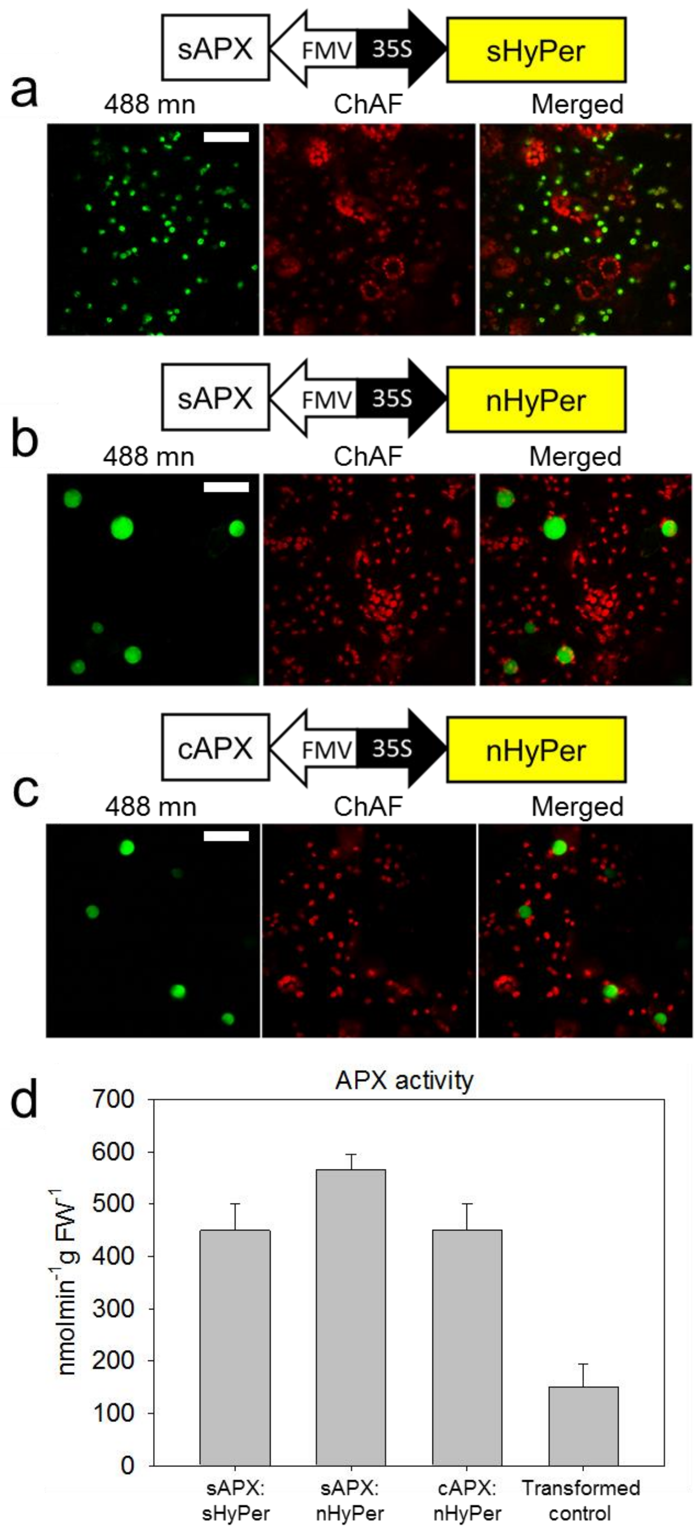

Supplementary Figure 6. Co-expression of sHyPer2 or nHyPer2 with sAPX and cAPX. (**a–c**) Images of the HyPer2 fluorescence from expression of the bicistronic constructs depicted above each figure confirming targeting of sHyPer2 to chloroplasts (a) and nHyper2 to nuclei (b-c). The columns of panels in (a-c) from left to right are the images of fluorescence excited by 480 nm excitation light, chlorophyll auto-fluorescence (ChAF) and merged images of the two. (**d**) APX activity in leaf cell free extracts from the plants co-expressing sAPX and sHyPer2, and sAPX and cAPX with nHyPer2 compared with an agro-inoculated control harbouring the Ti vector only (Transformed control). Scale bar = 50 µm.

Supplementary Table 1. Multi-model selection on generalised additive mixed effects models (GAMM). A range of models testing hypotheses on the effects of the X treatment (‘treat’) were fitted to the time series data for X; ‘treat’ assess differences in median values among the levels of the treatment, while comparisons between s(time) and s(time, by = treat) assess whether the shape of the time series differs among treatments. Models were compared via the small sample size corrected Akaike Information Criterion (AICc), delta AICc is the difference in AICc score relative to the model with the lowest value (most parsimonious model) and AICc Weight (Wt) is the relative support for the model. The best fitting models were selected as those returning the lowest AICc score and the highest AICc weight and are highlighted in bold.

Model	df	logLik	AICc	Delta	Weight
Cytosol					
**C1 – fixed = treat + s(time, by = treat)**	**8**	**-10.95**	**38.00**	**0.00**	**1.00**
C2 – fixed = treat + s(time)	7	-89.44	192.95	154.95	0.00
C3 – fixed = s(time, by = treat)	6	-98.69	209.44	171.44	0.00
C4 – fixed = s(time)	5	-167.63	345.30	307.30	0.00
C5 – fixed = 1	3	-221.91	449.84	411.84	0.00
Nucleus					
**N1 – fixed = treat + s(time, by = treat)**	**17**	**26.80**	**-18.19**	**0.00**	**0.74**
N2 – fixed = treat + s(time)	9	17.28	-16.14	2.04	0.26
N3 – fixed = s(time, by = treat)	5	-60.42	130.97	149.16	0.00
N4 – fixed = s(time)	13	-54.19	135.21	153.40	0.00
N5 – fixed = 1	3	-80.32	166.69	184.87	0.00
Stroma					
**S1 – fixed = treat + s(time, by = treat)**	**14**	**-2871.00**	**5770.23**	**0.00**	**1.00**
S2 – fixed = treat + s(time)	8	-2887.59	5791.25	21.02	0.00
S3 – fixed = s(time, by = treat)	11	-2930.33	5882.79	112.56	0.00
S4 – fixed = s(time)	5	-2946.20	5902.43	132.20	0.00
S5 – fixed = 1	3	-2956.93	5919.88	149.65	0.00

Supplementary Table 2. List of primers used in this study.

	primer name 5'→> 3'	Targeted/*underlined
P1	CGCaagcttGAAATGGCTTCTCAACAGG	HyPer ATG minus *HindIII
P2	TCACACTGCTTGCTTCAAAAC	HyPer (*stop codon)
P3	caccATGGCTTCCTCTATGCTCTC	RbcSF (Gateway directional cloning)
P4	caccATGCCTAAGAAGAAGAGAAAGGTTGTTTCCGTTATCGCAAAGC	NLS:HyPerF (*PKKKRKV)
P5	TCAAACCTTTCTCTTCTTCTTAGGTCTAGAACCACCAGAGCCAC	NLS:HyPerR (*PKKKRKV)
P6	GGAACATGGTAGCCGTTGGAAGTGGAATAA	SDM A233V (*nucleotides point mutation)
P7	TTATTCCACTTCCAACGGCTACCATGTTCC	SDM A233V (*nucleotides point mutation)
P8	AGGATGGACACTCCTTGAGGGATCAAG	SDM C199S (*nucleotide point mutation)
P9	CTTGATCCCTCAAGGAGTGTCCATCCT	SDM C199S (*nucleotide point mutation)
P10	caccATGGTTTCCGTTATCGCA	caccpHRedF
P11	TCATCTAGAACCACCAGAGC	pHRedR
P12	*GCGC*aagcttGTTTCCGTTATCGCAAAGC	pHRed ATG minus **Hind*III
P13	caccATGACGAAGAACTACCCAACC	APX1; At1g07890
P14	TTAAGCATCAGCAAACCCAAG	APX1; At1g07890
P15	caccATGGCAGAGCGTGTGTCTCTC	sAPX (At4g08390)
P16	TTAGATAACGATACCCTCCGG	sAPX (At4g08390)
P17	catcgttgaagatgcctctgc	CaMV35S promoter
P18	atcgagcagcagctggcttgtgg	FMV promoter
P19	GTCTGCTAGTTGTACGGAACC	HyPer
P20	GATGCCACTAAAGGCTCAGAC	Niben101Scf07050g00006 (NbAPXa)
P21	CAGCATAGTCAGCAAAGAAGG	Niben101Scf07050g00006 (NbAPXa)
P22	TTACTTTGACGTTGACATAGATGG	Nbcyc
P23	CACCTTGAAGCATGAAACTGG	Nbcyc
P24	GGGAGGCATGTTGTTGTTTCTC	NbZIP9
P25	AACTGATGGAACGACAAGGC	NbZIP9
